# Infrastructural requirements for local implementation of safety policies: the discordance between top-down and bottom-up systems of action

**DOI:** 10.1186/1472-6963-9-45

**Published:** 2009-03-09

**Authors:** Toomas Timpka, Cecilia Nordqvist, Kent Lindqvist

**Affiliations:** 1 Section of Social Medicine and Public Health, Department of Medicine and Health Sciences, Linköping University, SE-581 83 Linköping, Sweden

## Abstract

**Background:**

Safety promotion is planned and practised not only by public health organizations, but also by other welfare state agencies, private companies and non-governmental organizations. The term 'infrastructure' originally denoted the underlying resources needed for warfare, e.g. roads, industries, and an industrial workforce. Today, 'infrastructure' refers to the physical elements, organizations and people needed to run projects in different societal arenas.

The aim of this study was to examine associations between infrastructure and local implementation of safety policies in injury prevention and safety promotion programs.

**Methods:**

Qualitative data on municipalities in Sweden designated as Safe Communities were collected from focus group interviews with municipal politicians and administrators, as well as from policy documents, and materials published on the Internet. Actor network theory was used to identify weaknesses in the present infrastructure and determine strategies that can be used to resolve these.

**Results:**

The weakness identification analysis revealed that the factual infrastructure available for effectuating national strategies varied between safety areas and approaches, basically reflecting differences between bureaucratic and network-based organizational models. At the local level, a contradiction between safety promotion and the existence of quasi-markets for local public service providers was found to predispose for a poor local infrastructure diminishing the interest in integrated inter-agency activities. The weakness resolution analysis showed that development of an adequate infrastructure for safety promotion would require adjustment of the legal framework regulating injury data exchange, and would also require rational financial models for multi-party investments in local infrastructures.

**Conclusion:**

We found that the "silo" structure of government organization and assignment of resources was a barrier to collaborative action for safety at a community level. It may therefore be overly optimistic to take for granted that different approaches to injury control, such as injury prevention and safety promotion, can share infrastructure. Similarly, it may be unrealistic to presuppose that safety promotion can reach its potential in terms of injury rate reductions unless the critical infrastructure for this is in place. Such an alignment of the infrastructure to organizational processes requires more than financial investments.

## Background

Every year, intentional and unintentional injuries account for about five million deaths or 9% of all global mortality [1], the developing countries contributing almost 90% of this burden [2]. The injury mortality equals to the deaths from HIV, malaria and tuberculosis combined, and, correspondingly, injuries are recognized as one of the most important global public health problems. Several global trends converge to further aggravate the problem. For instance, road traffic accidents are in 2030 projected to have advanced to the 4^th ^place in the listings of single contributions to the global burden of disease [3]. There is therefore a pressing need for effective policies and programs addressing injury prevention and safety promotion. Even though these concepts often are used interchangeably, there are important theoretical and practical differences between them. Injury prevention denotes a scientifically informed "engineering" process focussed on scientists and experts, with prevention of motor traffic injuries as the model application area [4]. In contrast, safety promotion is planned and delivered not only by public health organizations, but also by other welfare state agencies, private companies, and civic non-governmental organizations in liaison with the general public [5,6]. In safety promotion, it is assumed that a top-down leadership of welfare state agencies can be coordinated with a bottom-up and problem-oriented mobilization of local community resources for a more pervasive prevention of injuries. To be able to plan such community-based programmes, the coordinators need to identify the relevant assets in the community, including resources in the private and non-government sectors. In other words, the provision of community-based safety promotion requires understanding and exploitation of the infrastructure, i.e. of the durable social, physical, and technical resources that are available in the community. Specifically, it needs to be understood how these resources can be accessed and employed in the local implementation of national policies and in community-derived programmes targeting the particular local safety issues. Even though a fundamental imbalance in power between community actors and representatives from various levels of government has been observed in numerous health promotion settings [7], infrastructural requirements have rarely been taken into consideration when designing and implementing different types of injury prevention and safety promotion programmes [8].

The term 'infrastructure' stems from 19th-century military vocabulary, in which it denoted the resources behind the front, necessary to wage war, i.e. structures such as roads, industries and railways, but also including an industrial workforce. In modern usage, 'infrastructure' includes both physical structures, such as water supplies and computer networks, and skilled professionals providing specific services [9]. In organizational settings, the infrastructure is most commonly seen as a utility aimed at reducing costs of processing and communicating materials and information throughout the organization [10]. Because the performance of key organizational processes depends on the infrastructure, the infrastructure is expected to be aligned with the organizational strategies, and vice versa.

The relationship between infrastructure and social development has been studied using several different theoretical models, ranging from the social construction of technology approach [11] to formal mathematical models [12]. One of these frameworks, which have been used to include also geographical aspects, is actor network theory [13,14]. In this theory, an actor network is defined by the non-technical and technical elements that influence action and decision-making in specific organizational or community settings. The theory is symmetrical in that it grants all elements of such heterogeneous networks the same explanatory status. In the case of safety promotion, an actor network therefore literally includes the welfare state agencies, companies, populations, policy-makers, as well as a diverse set of linked physical structures, products, legislations, and other materials that are involved in or used for prevention of injuries.

The aim of this study was to examine associations between infrastructure and local implementation of safety policies in injury prevention and safety promotion programs. Actor network theory was first used to represent weaknesses in the present infrastructure, and thereafter to analyse intervention strategies that can be used to resolve these weaknesses. Data for the analyses were collected on the implementation of 'Safe Community' programmes in Swedish municipalities.

## Methods

Qualitative research methods were employed for data collection and analysis. Specifically, a case study design [15] based on comparisons between multiple parallel cases (municipal programmes) was used to perform an actor network analysis of the infrastructure underpinning community-based safety promotion. The Regional Ethical Review Board in Linköping approved the study protocol before data collection. Qualitative data were collected from focus group interviews with municipal politicians and administrators, as well as from policy documents, and materials published on the Internet. The concept of global and local actor networks introduced by Callon and Law [16] was used to structure the analyses. According to these authors, organizational actors form a *global actor network *in order to obtain resources with which to achieve prevalent goals and objectives. This network is a socio-technical web that both generates processes of planning and uses different degrees of 'remote control' to implement actions in *local actor networks*. To be able to analytically integrate the global and local networks, equal attention in actor network analysis therefore needs to be paid to the micro-level local processes, on the one hand, and the macro-level global structures, on the other.

In the first phase, researchers with a background in safety promotion and the social sciences analysed the data to create an actor network theory of the present situation. Thereafter, additional public health researchers joined the actor network analysis to identify interventions that would support the establishment of an aligned infrastructure for community-based safety promotion.

### Data collection

Focus group interviews were performed with representatives from 10 Swedish municipalities which between 1989 and 2000 had been declared as being a Safe Community according to the criteria [17] stated by the WHO Collaboration Centre for Safe Communities. In order to obtain data from subjects with long experience of safety promotion work, the ten first designated Safe Communities in Sweden were invited to participate in the study. All communities, agreed to participate. The communities were different types of municipalities; four were medium-sized, two sparsely populated one suburban one industrial and two large municipalities. All regions of Sweden were represented, with a concentration to the south-west region. The research team planned four focus group interviews with five participants in each session. One local government administrator from each of five different municipalities was invited to participate in the first session. To the second session, one local government politician from each of the same five municipalities was invited. The third and fourth sessions were planned in the same way. Administrator and politician sessions were organized in groups of five persons, with one representative from each of the other five municipalities participating in each session. The interview guide contained questions ranging from how the subjects defined community-based safety work to perceived problems and opportunities.

In March 2007 CN moderated the four interviews. All administrators and six out of ten politicians invited to attend the two politicians' group interview sessions participated. Shortly after these sessions, the moderator conducted individual telephone interviews with the remaining four politicians. The semi-structured interviews were audio-recorded, and transcribed verbatime. At the same time, policy documents from the municipalities and the national agencies working with safety issues were collected. In addition, the websites of the municipalities and agencies were scanned for further documentation on safety policies and ongoing programmes.

### Data analysis

A qualitative content analysis of the data from the interviews and documents was performed, focusing on the manifest content [18]. The first stage of the analysis, the analysis of the present situation, was initiated by examination of an actor network for community-based safety promotion. A global actor network was defined as one that secures the technical and relational resources that are necessary for implementation of activities at an operational level. The analysis was then turned to the micro level and the dataset was examined to identify themes and explore categories of micro-level issues in safety promotion practice. A 'local network actor' was defined as one that uses the resources provided by a global actor network to develop particular networked organizational processes for local safety promotion.

Thereafter, the second part of the analysis was initiated by validating the network descriptions at both levels and relating them to national and local service policy documents. The last phase of the investigation of the present situation focused on integrating the micro- and macro-level analyses. Correspondingly, specific attention in this phase was paid to the points of passage between the global and local networks. Preliminary results were presented as both text and graphics, discussed, and iteratively revised. Observations made by the different researchers were compared, discussed and integrated in order to identify specific implications for the infrastructure.

At the second stage of the analysis, the actor network representation was used to identify interventions that would enable the establishment of an aligned infrastructure for community-based safety promotion. For each problem area, a set of possible interventions with the potential to reduce obstructions and difficulties were identified. From this set, the most realistic intervention within a 5-year perspective was chosen for representation in a hypothetical, reconstructed actor network. The reliability of the analyses was tested using informal examination of the intra-rater reliability; the first author tested the results by repeating parts of the analyses on other injury areas than those reported. The inter-rater reliability was not tested, but the results were continuously discussed and commented upon among the authors. The results were also reviewed and validated by a group with extensive experience from safety research and practice, consisting of representatives (n = 9) from the Swedish National Board of Health and Welfare, Karolinska Institutet in Stockholm, the Swedish National Institute of Public Health, and Umeå University.

## Results

In Sweden at the time of data collection, most costs for public health and welfare services were financed by income taxation and organized in 'quasi-market' systems, where Minister-led national government Departments regulated the competition for local government contracts between service providers. This situation was true also for the agencies involved in injury prevention and safety promotion. Another basis for the activities in the safety area was the Civil Protection Act (2003:778). This legislation addresses the entire safety management process from injury prevention to emergency response and post-event measures, and specifies the responsibilities of the individual as well as those of the local and national governments. The Act stipulates that general objectives for civil protection be formulated by the national agencies for emergency management and rescue services, and that municipalities establish action programmes for local civil protection including both injury prevention and emergency response.

### Global (national) safety actor networks

**Figure 1:**
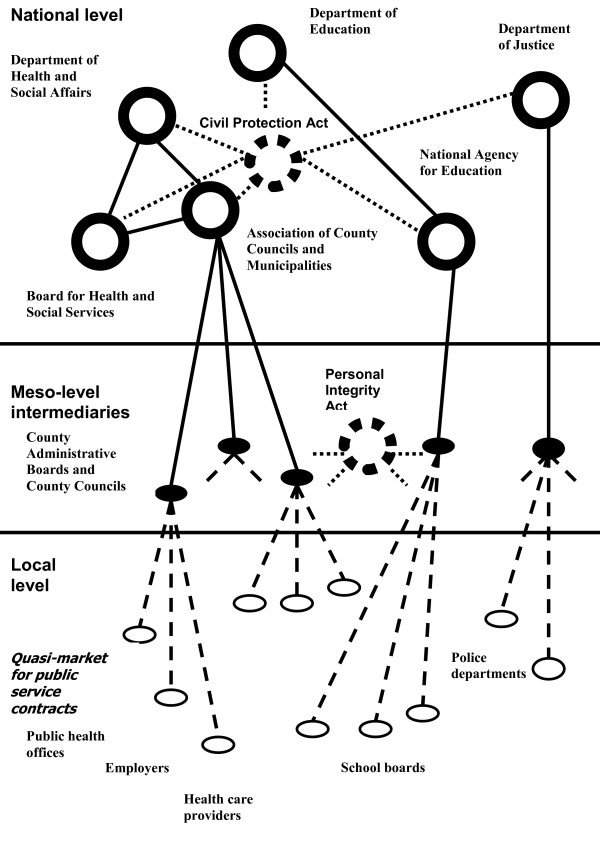
**Generalized structure of the actor network for safety promotion, observed at the time of the study**. The global network was divided into sub-networks with conflicting goals and agendas. The quasi-market for public service contracts led to a situation where the organizational support for safety promotion activities from local agencies was fragmentary and inconsistent. Legislation (the Civil Protection Act and the Personal Integrity Act) is shown by dotted lines.

We found that the factual infrastructure available for effectuating national safety strategies varied considerably between different safety areas and organizational levels, basically as a consequence of the hierarchical structure of the national government administration. In some areas, such as traffic safety, there was an alignment between the national, regional and local agency levels, allowing distribution of strategies and resources to the local levels and a return of data from local injury surveillance. However, in other safety areas the welfare state agencies that were expected to cooperate at the local level to implement national directives were part of separate actor networks. In consequence, several safety areas, such as school and home safety, lacked a shared infrastructure for safety management. For instance, regarding school safety, the local public health service resources were managed by the county councils, while the school building maintenance resources were managed by the municipal councils, and law enforcement was managed by the regional police boards. These agencies were in turn managed by different national government Departments and agencies (Figure 1). The local implementation of national-level safety directives was therefore not always straightforward. As an example, at the time of the study, drowning was recognized by the National Rescue Services Agency as a major safety problem among children. The national Department of Health and Social Affairs recommended that all children should learn to swim in elementary school. However, while this government Department had promotion of health on an equal basis to the population on its agenda, it was the Department of Education that was responsible for the school curricula. The Minister of Education was reluctant to add new items to an already full agenda for the children, and the Department of Education correspondingly did not agree to introduce swimming training programmes in elementary schools. The consequence was that the local safety promotion coordinators received contradictory directives regarding the prevention of drowning among children from two different national government bodies, the Department of Health and Social Affairs and the Department of Education. These Departments had substantially dissimilar operational objectives, and the Ministers in charge had different political agendas. This situation, where a safety problem was identified in one government jurisdiction area and needed to be addressed in another, was not restricted to the Departments of Health and Education. Despite this fact, there was no mechanism in place that prevented national-level safety and injury prevention initiatives in one area from being neutralized by welfare state ambitions or reforms in other areas.

### Local safety actor networks

A different set of problems were observed in association with municipalities mobilizing networks for addressing local injury problems. The Civil Protection Act can be interpreted as an expression of the 'new public health' model, which emphasizes local development of health-promoting environments. This model was broadly introduced in the 1990s. However, in Sweden, quasi-markets aimed at improving the effectiveness of the welfare state bureaucracy were introduced almost at the same time, the aim being that the welfare state agencies and their sub-departments should compete for government contracts. This meant that, on the one hand, the welfare state agencies were anticipated to collaborate with each other in the administration of safety promotion programmes, while on the other they were pushed apart by the (quasi) market forces. In particular, the existence of local quasi-markets meant that middle managers responsible for budgets at local public service offices found little incentive for cooperation across organizational boundaries. Similarly, there was little interest in building a shared infrastructure in support of local safety promotion, e.g. by implementation of local injury surveillance systems. It was mainly the municipalities that needed the injury data in order to plan and implement preventive measures, while the collection and analysis of the data was the responsibility of the public health offices managed by the county councils. The contradiction between the safety promotion and quasi-market paradigms therefore predisposed for a poor, or lacking, local infrastructure diminishing the interest in agreements about inter-agency sharing of surveillance data and integrated evaluations of interventions. These difficulties were associated not only with separation between the national-level departments and agencies, but also with differences in the geographical jurisdiction areas between the local offices that needed to collaborate. For instance, the public health office responsible for injury surveillance was managed at the county level, and therefore had to coordinate their processes with between six and ten municipal councils, each formally responsible for safety promotion among their residents.

#### Towards an adjusted infrastructure for safety promotion

**Figure 2:**
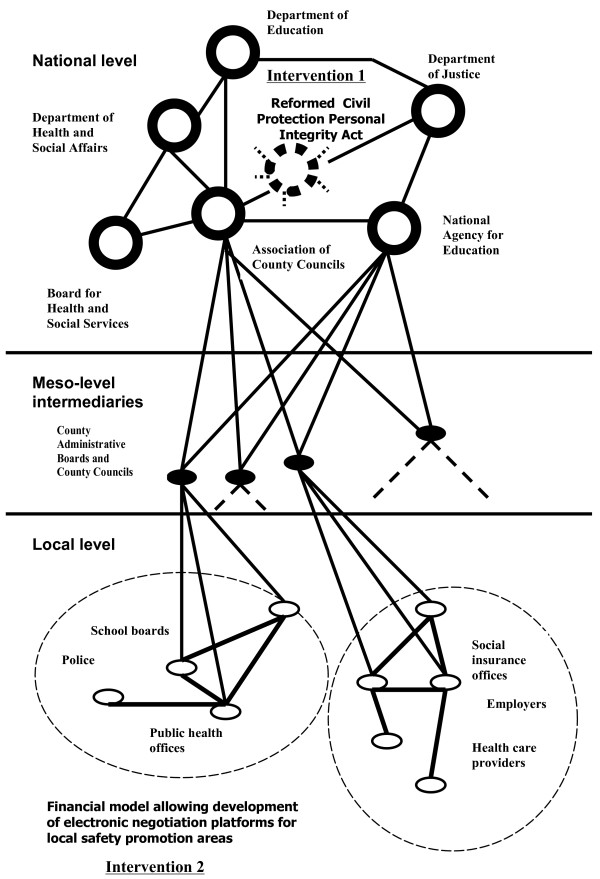
**Generalized structure of a reformed actor network for community-based safety promotion**. Two interventions are shown in the reformed actor network, (1) a common legal framework allowing sharing of injury data; and (2) financial models allowing local welfare state offices to develop a shared infrastructure for safety promotion in the community.

The intervention step in the actor network analyses revealed two areas where the infrastructure for community-based safety promotion could be adjusted. These areas were the legal framework regulating injury data exchange, and financial models for investments in negotiation spaces for locally important safety areas. Consequently, two corresponding organizational interventions necessary for the development of sustainable community-based safety promotion services were identified (Figure 2).

### Legal frameworks supporting safety promotion

Introduction of new health policies requires empirical validation of the problem and support among those in a position of strategic power [19]. Both these prerequisites depend on the collection, analysis, and presentation of data – in other words, the welfare state agencies involved in safety promotion need to collect and share injury data at both the local and the national level. In our example, the coordinators of local safety promotion programmes needed information about the injury patterns in the community, even though they did not need to know details of every individual injured person's background, and the medical character of their injuries. However, the Swedish Personal Integrity Act does not permit transfer of client data between welfare state agencies. In other words, at the time of the study there was in Sweden a contradiction between the Civil Protection Act and the Personal Integrity Act in its endeavour to protect the citizens. To resolve this dilemma, a modern legal framework for sharing of safety-related data is needed, which still does not threaten the individual citizen's integrity.

In Sweden, the call for a legal framework allowing efficient data interchange in health care has recently been highlighted and acted upon. A national computer-based infrastructure for health information management has been proposed, based on an integrated strategy for health care delivery and supply of social welfare services [20]. The public and private sectors are expected to collaborate in building this infrastructure, which is essentially a 'paperless' national social welfare administration. It is recognized that a central issue in this development is addressing the legal issues and protecting the integrity of the individual citizen. From the safety promotion perspective, it is essential that such a renewed legal framework is not restricted to public health and health care organizations alone, but incorporates the agencies and actors included in the Civil Protection Act. Opportunities for balancing scientifically validated legal frameworks against the priorities of political leaders, social welfare organizations, commercial interests, and the public opinion must therefore be systematically exploited, and made available over time. This can only be achieved where there is an effective interaction between those at the relevant social welfare agencies with insights into the injury problem and those among policy-makers with a mandate for taking action. This alignment is only possible to achieve at national government level.

### Financial models for investments in a local safety infrastructure

The safety promotion programmes included in this case study were implemented in comparatively small communities. Physical meetings, restricted both by their duration and intervals, constituted the *negotiation space *where inter-sector exchange of experiences took place and strategic decisions were made. However, using modern information and communication technology, these processes could continue also in the interval between physical meetings. In health care settings, a networked local electronic patient record allowing sharing of data and experiences on frequent out-patient care users has been reported to improve the efficacy and quality of services [21]. Nevertheless, while health care services are provided within one welfare state sector, safety promotion is an inter-sector enterprise. For local electronic negotiating platforms to be developed for safety promotion, these have to be organizationally and financially supported by the offices involved. Today the technical and organizational methods for implementation of such inter-organizational systems are available, including procedures for integration of computer networks and methods for introducing the system to different staff categories [22,23]. The obstacle is, however, a lack of models for financing the local development [24]. In the case study setting, the quasi-market system made it difficult for the welfare state offices to contribute capital for investments in local infrastructure. Experiences from the US and EU suggest that national government subsidies and loans need to be combined with formation of local public-private partnerships for funding infrastructural development in local communities [25]. The formation of these partnerships requires careful planning. Economic success (being wealthy and materially successful) has in experiments been shown to be placed in the external-physical end of the human motivational system, and in direct opposition to community-feeling (community support through activism or generativity) which is placed in the intrinsic and self-transcendental end [26,27]. It is thus not straight-forward to combine short-term economic and community motives when pleading for investments in local infrastructure. The rationale for subsidising local infrastructure for safety promotion should therefore be centred on long-term advantages, where material and immaterial short-term motives can be integrated using higher level values and objectives. Nevertheless, it must also be remembered that investments in infrastructure do not guarantee local safety policies to succeed; they only provide the necessary basis for the possibility of success.

## Discussion

Physical elements (natural or constructed), people, and organizations are grouped in different ways to develop critical infrastructure for different societal areas [9]. The aim of this study was to examine associations between infrastructure and local implementation of safety policies in injury prevention and safety promotion programs. We found a higher-order dissimilarity between two generic approaches to public service provision, i.e. the hierarchical 'Weberian' bureaucracies (such as government agencies for road injury control), and distributed and networked organizations working in collaboration with civic groups and citizens, represented in this study by the local safety promotion programmes. While the bureaucratic injury control programmes address the injury problem by using rational-technical interventions directed at specified problems, the networked safety promotion programmes focus on awareness and enhancement of safety-related behaviours at the community level, and enforcement of social relations supportive of this task. In the study setting, it was never questioned whether the municipalities obliged to effectuate safety promotion under the Civil Protection Act had an infrastructure in place comparable to that available for the bureaucratically organized agencies dealing with safety problems, e.g. the agency for road injury control. Our findings suggest that the infrastructural support for community-based safety promotion was neither adequate, nor was it able to meet the requirements posed upon these programmes. It may therefore be overly optimistic to take for granted that the two approaches to injury control, also in other settings, can be assumed to rely only on the available infrastructure. Similarly, it may be unrealistic to presuppose that safety promotion can reach its potential in terms of injury rate reductions unless the critical infrastructure for this is in place. Such an alignment of the infrastructure to organizational processes requires more than financial investments. For instance, we found that negotiation about adjustments of legislation and re-organization of the government administration at the national and local levels is equally important. Using similar social network methods for their analyses [28], also Australian researchers have reported from studies of local safety work that resources (and infrastructure) are essentially controlled by external government agents. This is of course how government bodies maintain control over the social agenda. Therefore, an important strategic skill of community leaders representing safety promotion programmes is their ability to personally connect with government bodies and navigate the tension between the two different paradigms of social organization (informal community networks and formal hierarchical systems).

In support of the alignment of programme processes with underlying resources in safety promotion, we advocate that researchers and practitioners in the area should follow the lead from health geography [29] by recognizing that a multitude of reciprocal relationships may exist between humans and their environment, which affect health outcomes. Scientific methods and intervention planning procedures used for safety promotion should therefore be extended to allow representation of relational systems and models. Using such a perspective, our actor network analyses showed that the bureaucratic injury control organizations and the networked safety promotion programmes had developed 'spaces of prescription' and 'spaces of negotiation', respectively, at the local level [30]. The concept of 'space' is here used to mean 'arrangements of priorities' [31]. Accordingly, 'prescription' and 'negotiation' highlight the interactions which take place as certain safety priorities, such as those emerging from implementing national injury control polices, compete with others, e.g. those emerging from efforts to establish local safety promotion networks. The relations in the spaces of prescription have relatively fixed positions and tend to be marked out by standardized datasets, while the spaces of negotiation are distinguished by fluidity and variation as actors or coalitions of actors come together to negotiate their memberships and affiliations. We believe that models based on this type of relational approach are necessary for understanding how infrastructure is associated with reduction of injuries in safety promotion, and that such descriptions are also a prerequisite for planning investments and changes in safety policies that are both effective and contextually sensitive.

Our qualitative study was performed in a specific setting, a highly industrialized country with a long democratic tradition and an extensive social welfare system. The results can therefore not be directly applied to other socio-cultural and economic settings. However, there are aspects in our findings that can be interpreted as being of importance for safety promotion programmes in other settings. While an infrastructure for a 'Weberian' hierarchical government administration has been established in most developed and developing countries, the infrastructure available for well-distributed safety promotion programmes can be assumed to be quite different. For instance, the possibilities to provide for local negotiation spaces shared between local government offices, welfare state agencies, and civic organizations, where local injury data can be collected and analysed and interventions decided upon, differ considerably both with level of urbanization and with the basic information infrastructure of a region. These observations need to be taken into consideration both when introducing safety promotion programmes in new regions, and when evaluating implemented programmes.

## Conclusion

We found that the "silo" structure of government organization and assignment of resources was a barrier to collaborative action for safety at a community level. It may therefore be overly optimistic to take for granted that different approaches to injury control, such as injury prevention and safety promotion, can share infrastructure. Similarly, it may be unrealistic to presuppose that safety promotion can reach its potential in terms of injury rate reductions unless the critical infrastructure for this is in place. Such an alignment of the infrastructure to organizational processes requires more than financial investments.

## Competing interests

The authors declare that they have no competing interests.

## Authors' contributions

TT conceived of the study, participated in its design and coordination, and drafted the manuscript. CN carried out the data collection, participated in the study design and coordination, and helped to draft the manuscript. KL participated in the study design and coordination, and helped to draft the manuscript. All authors read and approved the final manuscript.

## Pre-publication history

The pre-publication history for this paper can be accessed here:


